# A67 ABC, EASY AS 123, SIMPLE AS GLUTEN-FREE? KNOWLEDGE AND ADHERENCE IN ADOLESCENTS WITH CELIAC DISEASE

**DOI:** 10.1093/jcag/gwab049.066

**Published:** 2022-02-21

**Authors:** K Pohoreski, D Gidrewicz

**Affiliations:** University of Calgary, Calgary, AB, Canada

## Abstract

**Background:**

Research shows that adolescents with celiac disease (CeD) are at great risk for non-adherence to the gluten-free diet (GFD), and that knowledge of the GFD may play a role in adherence.

**Aims:**

The primary objective of this study was to examine the relationship between patient knowledge and adherence to the GFD in a local population of adolescents with CeD. Secondary objectives were to identify information sources used to learn about the GFD, and compare adolescent and parent knowledge of the GFD.

**Methods:**

Adolescents with CeD (12–17 years) were recruited at pediatric gastroenterology clinics in Calgary, Alberta. Preliminary data was collected October 2020 to August 2021. Both the adolescent and a parent were asked to complete a 5 minute online survey received via email. Survey measures included a validated adherence scale (Biagi score) and a unique knowledge assessment (GFD-Q), adapted from a previously published questionnaire. GFD-Q scores were categorized as “sufficient knowledge” based on correctly identifying 3/3 gluten-containing foods, at least 4/7 of gluten-free foods, and at least 4/7 foods that may contain gluten. Respondents submitted demographic and clinical information, as well as reviewed information sources used to learn about the GFD.

**Results:**

Initial data were reviewed for 25 participant pairs (adolescent with respective parent), with a planned sample size of 40 participant pairs. Most adolescents (96%) reported adherence to the GFD (Biagi score of 3 or 4). Eleven (44%) adolescents had sufficient knowledge of the GFD. About 90% of adolescents correctly identified which foods to question, however, 20% did not correctly identify a gluten-containing food (namely malt), and 36% did not identify most gluten-free foods (such as maltodextrin and balsamic vinegar). Parents scored higher on the GFD-Q with 17/25 (68%) demonstrating sufficient knowledge. Interestingly, of the 11 adolescents with sufficient knowledge, 10/11 (91%) respective parents also had sufficient knowledge. Most adolescents and parents identified the gastroenterologist, another individual with CeD, and Google as helpful information sources. Adolescents also indicated their parents were a helpful information source.

**Conclusions:**

While the study is still enrolling to reach sample size, we acknowledge that most adolescents in our study report being adherent to the GFD. However, their reported knowledge is variable, especially in knowing which foods are safe to eat. Thus, education should also focus as much on which foods to avoid, as well as safe foods to consume. Parent knowledge of the GFD may have a role in adolescent knowledge. At study completion, descriptive statistics will be used to examine participant characteristics and identify trends to assist in the development of future health education for adolescents with CeD.

**Funding Agencies:**

Canadian Celiac Association - James A.Campbell Research Fund

